# AI-Based Identification Method for Cervical Transformation Zone Within Digital Colposcopy: Development and Multicenter Validation Study

**DOI:** 10.2196/69672

**Published:** 2025-03-31

**Authors:** Tong Wu, Yuting Wang, Xiaoli Cui, Peng Xue, Youlin Qiao

**Affiliations:** 1 School of Population Medicine and Public Health Chinese Academy of Medical Sciences and Peking Union Medical College Beijing China; 2 Liaoning Cancer Hospital and Institute Department of Gynecologic Oncology Cancer Hospital of China Medical University Shenyang China

**Keywords:** artificial intelligence, AI, cervical cancer screening, transformation zone, diagnosis and early treatment, lightweight neural network

## Abstract

**Background:**

In low- and middle-income countries, cervical cancer remains a leading cause of death and morbidity for women. Early detection and treatment of precancerous lesions are critical in cervical cancer prevention, and colposcopy is a primary diagnostic tool for identifying cervical lesions and guiding biopsies. The transformation zone (TZ) is where a stratified squamous epithelium develops from the metaplasia of simple columnar epithelium and is the most common site of precancerous lesions. However, inexperienced colposcopists may find it challenging to accurately identify the type and location of the TZ during a colposcopy examination.

**Objective:**

This study aims to present an artificial intelligence (AI) method for identifying the TZ to enhance colposcopy examination and evaluate its potential clinical application.

**Methods:**

The study retrospectively collected data from 3616 women who underwent colposcopy at 6 tertiary hospitals in China between 2019 and 2021. A dataset from 4 hospitals was collected for model conduction. An independent dataset was collected from the other 2 geographic hospitals to validate model performance. There is no overlap between the training and validation datasets. Anonymized digital records, including each colposcopy image, baseline clinical characteristics, colposcopic findings, and pathological outcomes, were collected. The classification model was proposed as a lightweight neural network with multiscale feature enhancement capabilities and designed to classify the 3 types of TZ. The pretrained FastSAM model was first implemented to identify the location of the new squamocolumnar junction for segmenting the TZ. Overall accuracy, average precision, and recall were evaluated for the classification and segmentation models. The classification performance on the external validation was assessed by sensitivity and specificity.

**Results:**

The optimal TZ classification model performed with 83.97% classification accuracy on the test set, which achieved average precision of 91.84%, 89.06%, and 95.62% for types 1, 2, and 3, respectively. The recall and mean average precision of the TZ segmentation model were 0.78 and 0.75, respectively. The proposed model demonstrated outstanding performance in predicting 3 types of the TZ, achieving the sensitivity with 95% CIs for TZ1, TZ2, and TZ3 of 0.78 (0.74-0.81), 0.81 (0.78-0.82), and 0.8 (0.74-0.87), respectively, with specificity with 95% CIs of 0.94 (0.92-0.96), 0.83 (0.81-0.86), and 0.91 (0.89-0.92), based on a comprehensive external dataset of 1335 cases from 2 of the 6 hospitals.

**Conclusions:**

Our proposed AI-based identification system classified the type of cervical TZs and delineated their location on multicenter, colposcopic, high-resolution images. The findings of this study have shown its potential to predict TZ types and specific regions accurately. It was developed as a valuable assistant to encourage precise colposcopic examination in clinical practice.

## Introduction

Cervical cancer remains the fourth most prevalent cancer among women worldwide [1], and it continues to be a leading cause of morbidity and mortality threatening women’s health. Since cervical precancerous lesions often progress to invasive cancer over an extended period, early detection is critical for cervical cancer prevention. Colposcopy serves as a crucial component of cervical cancer screening, providing a preliminary diagnosis for patients based on screening results, which then guides subsequent biopsy and treatment. Although it is a fundamental technique that health care providers can easily teach and implement, the strong subjective nature of colposcopy diagnosis makes it difficult for colposcopists with different qualifications to perform standardized diagnoses and make effective clinical decisions [2,3]. Artificial intelligence (AI) diagnostic technology could resolve the disparities in expertise among clinicians and enhance screening efficiency [4].

The transformation zone (TZ) is where a stratified squamous epithelium develops from the metaplasia of simple columnar epithelium and is the most common site of precancerous lesions. More than 90% of cervical cancers develop within the TZ [5], making it a critical region for cervical intraepithelial neoplasia (CIN) diagnosis and early treatment. According to the visibility of the squamocolumnar junction (SCJ), the TZ can be classified into three types: TZ1 (SCJ fully visible), TZ2 (SCJ fully visible under endocervical speculum), and TZ3 (SCJ partially visible or not visible) [6]. Accurately identifying the TZ is crucial for diagnosing and treating cervical precancerous lesions. As the TZ moves into the cervical canal with increasing age, endocervical curettage (ECC) is necessary for biopsy-guided pathology [7]. If TZ types are not classified, the importance of ECC for the canal may be neglected, leading to missed diagnosis of lesions during colposcopic examination. In addition, excision of the entire TZ is a standard treatment for cervical precancerous lesions. For excisional treatment, TZ types determine the length and depth of the cervix to be excised. In destructive treatments, a prerequisite is that the TZ must be either type 1 or type 2. Therefore, the type and location of the TZ are the determinants of treatment choices, and accurately assessing the TZ is essential for guiding more effective biopsies and precise treatment.

However, in underserved population, the skills of colposcopists are generally suboptimal, with colposcopic finding accuracy being significantly lower than desired [8,9]. AI-assisted technology could effectively enhance the competencies of colposcopists in these underserved areas. Current evaluation studies [10,11] have demonstrated that junior or less-experienced colposcopists can detect abnormal cervical lesions more effectively with AI assistance, which indicates its potential to help reduce missed diagnoses. However, the functions of AI cannot be limited. All colposcopic features were the indicators for assessing colposcopist performance. Among them, the accurate identification of TZ types is essential for implementing effective colposcopy diagnostics and treatment procedures, potentially reducing the number of missed diagnoses and unnecessary biopsy procedures. However, current AI-assisted colposcopy systems or developed AI diagnosis models do not include TZ or SCJ detection during model conduction. Therefore, it is essential to integrate important clinical features into AI model training to improve colposcopy diagnosis efficiency.

AI colposcopy diagnostic systems remain challenging to distinguish among benign, CIN1, CIN2, and CIN3+ cases during colposcopy examination [12-14], although they achieved over 80% accuracy in detecting high-grade squamous intraepithelial lesions. This difficulty is attributable to the lack of specificity in the CIN-related acetowhite staining characteristics. Typically, normal cervical features, such as immature squamous metaplasia, congenital TZ, inflammation, and epithelium regeneration, may exhibit mild acetowhite reactions similar to those associated with CIN. This similarity implies that relying solely on acetowhite area features can easily lead to misclassification either for AI or in less experienced colposcopist. In AI model training, standardized annotated images of different acetowhite morphologies or fine-grained lesion descriptions may help with a more precise assessment of acetowhite characteristics [15]. However, current AI-assisted colposcopy research is constrained by the lack of standardized annotated colposcopy images. From a clinical perspective, the colposcopy guidelines issued by the International Federation for Cervical Pathology and Colposcopy (IFCPC) emphasize that CIN-related acetowhite changes are most commonly found in the TZ, and near the new SCJ, with clear demarcation from the surrounding epithelium [6]. Therefore, the TZ region can be used as an indicator to identify lesion areas and can be developed as a learned feature for diagnostic model development, thereby resolving the problem of the lack of annotated colposcopy images. As a result, the multiclassification accuracy of AI-guided colposcopic diagnostic systems may be significantly improved. Therefore, in AI model development, accurately identifying TZ types and the SCJ is a crucial step toward improving AI diagnostic accuracy and guiding biopsies.

In this study, an AI method is developed and validated for the classification and delineation of the TZ. This method not only has the potential to guide clinical colposcopic examinations in resource-limited health care settings but also encourages the advancement of AI-guided digital colposcopic systems. By incorporating all colposcopic findings, such as TZ type and features of both minor and major lesions, AI-guided digital colposcopy could become a mature assistant for universal clinical colposcopy examinations.

## Methods

### Study Patients

This retrospective study included 3616 women who underwent colposcopy examinations across 6 multicenter hospitals in China between January 2019 and October 2021. These hospitals were the Gansu Maternity and Child Healthcare Hospital, Second Hospital of Shanxi Medical University, Shenzhen Maternity and Child Healthcare Hospital, Jiangxi Maternity and Child Healthcare Hospital, The Affiliated Hospital of Qingdao University, and Chengdu Women’s and Children’s Central Hospital. The digital clinical records, including cytology, human papillomavirus infection status, colposcopy findings, and pathological results, were collected. All colposcopy images were captured using digital high-definition video colposcopes (Zonsun Healthcare Co Ltd, Edan Instruments). Colposcopy findings, including adequacy, SCJ visibility, TZ determination, and provisional diagnosis, were qualified by colposcopy experts from tertiary hospitals. The “ground truth” for TZ classification and SCJ visibility in this study was determined by an expert panel following IFCPC guidelines.

The inclusion criteria were as follows: (1) women with complete colposcopy findings and pathological diagnosis, (2) ages between 24 and 65 years, and (3) each record containing at least 5 satisfactory colposcopic images before and after acetic acid staining. The exclusion criteria were (1) all saline solution images (preacetic), since the TZ typically appears after acetic acid staining; (2) poor-quality images, such as overexposed images or those where the cervix was obscured or there was bleeding after the biopsy; (3) inadequacy colposcopic examination; and (4) records with missing TZ types or SCJ visibility. For external validation, an independent dataset was derived from The Affiliated Hospital of Qingdao University and Chengdu Women’s and Children’s Central Hospital. The training dataset was divided into training and test sets in an 8:2 ratio, with 10% of the training set used for validation during model tuning.

### Transformation Zone

The colposcopy procedures adhered to the standard guidelines for *Colposcopy and Treatment of Cervical Precancer* [6]. Time-series images were captured for each case, including 1 original cervix image (saline solution) and at least 4 acetic acid–stained cervix images. The classification of TZ followed guideline criteria. TZ was classified as type 1 when it was entirely located on the ectocervix. In type 2, TZ was partially located within the endocervical canal, but its upper limit could be visualized using auxiliary instruments. Type 3 TZ lies partly or entirely inside the endocervical canal, with its upper limit being partially or completely invisible, even with the aid of auxiliary instruments.

### Image Preprocessing

**Figure 1:**
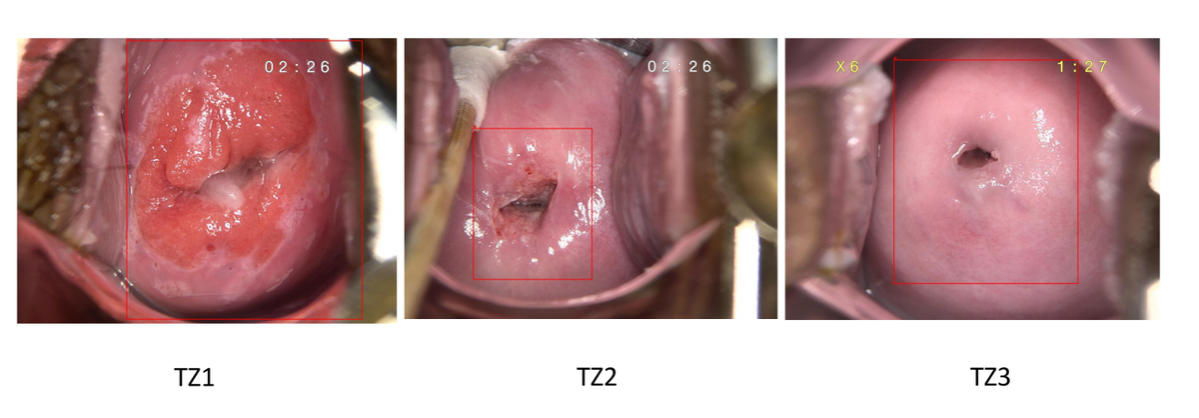
Cervix region of interest detection with bounding box examples in representative images. TZ: transformation zone.

The colposcopy images were captured in high resolution. However, they contained irrelevant objects, such as the endocervical speculum, cotton swabs, or large regions of the vaginal wall, which could interfere with the classification model’s ability to extract critical features of the cervix. To address this issue, we used the YOLOv5 network to detect the region of interest. Its single forward pass design enables real-time object detection with high efficiency and precision [16,17]. The integrated cervical region was automatically segmented and examined by a specialist (Figure 1). It divides an image into an S×S grid cell, predicting the bounding box locations and their associated categories. All segmented colposcopy images were resized to 224×224 pixels to align with the input specifications of the model.

### Development of the TZ Classification Model

**Figure 2:**
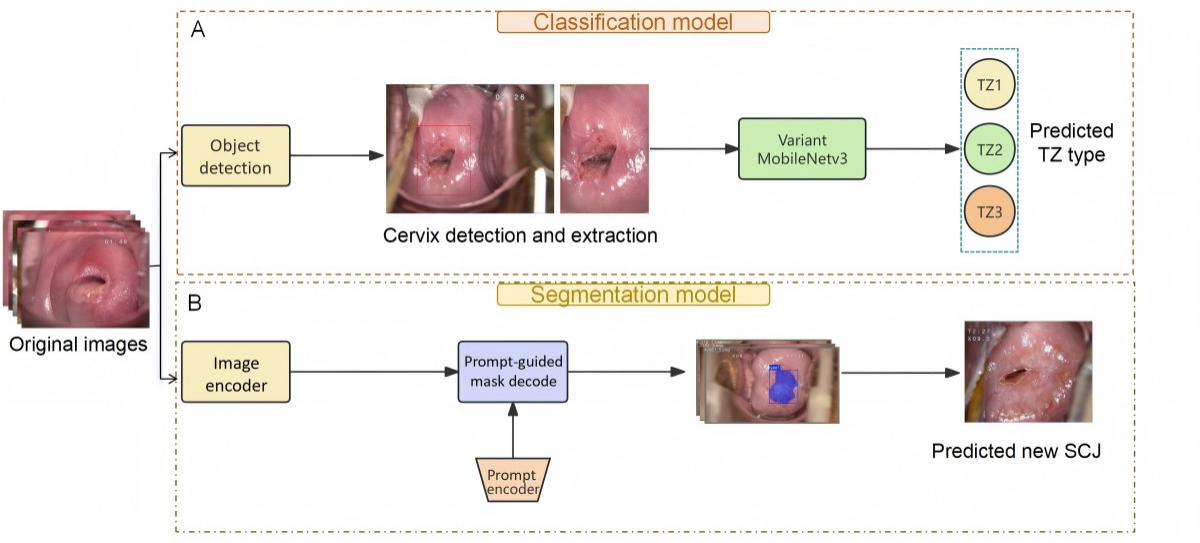
The overall inference process of the TZ identification system consists of two stages: (A) detection and extraction of the entire cervical region from original images, followed by feature extraction using the variant MobileNetV3 and inference TZ1, TZ2, and TZ3 types; and (B) using the original images and images with polygon of the new SCJ as prompts to FastSAM, which then outputs the mask prediction of the out-of-TZ area to infer the new SCJ guidance line. SCJ: squamocolumnar junction; TZ: transformation zone.

Initially, colposcopy images were input into a detection model to determine the cervical region, which was then preprocessed to enhance feature extraction. These data were then applied to a classification model to determine the types of TZ present (Figure 2A). In the second part of the method, the original images were annotated with new SCJ prompts to guide a general segmentation model in inferring the potential location of the TZ (Figure 2B).

**Figure 3:**
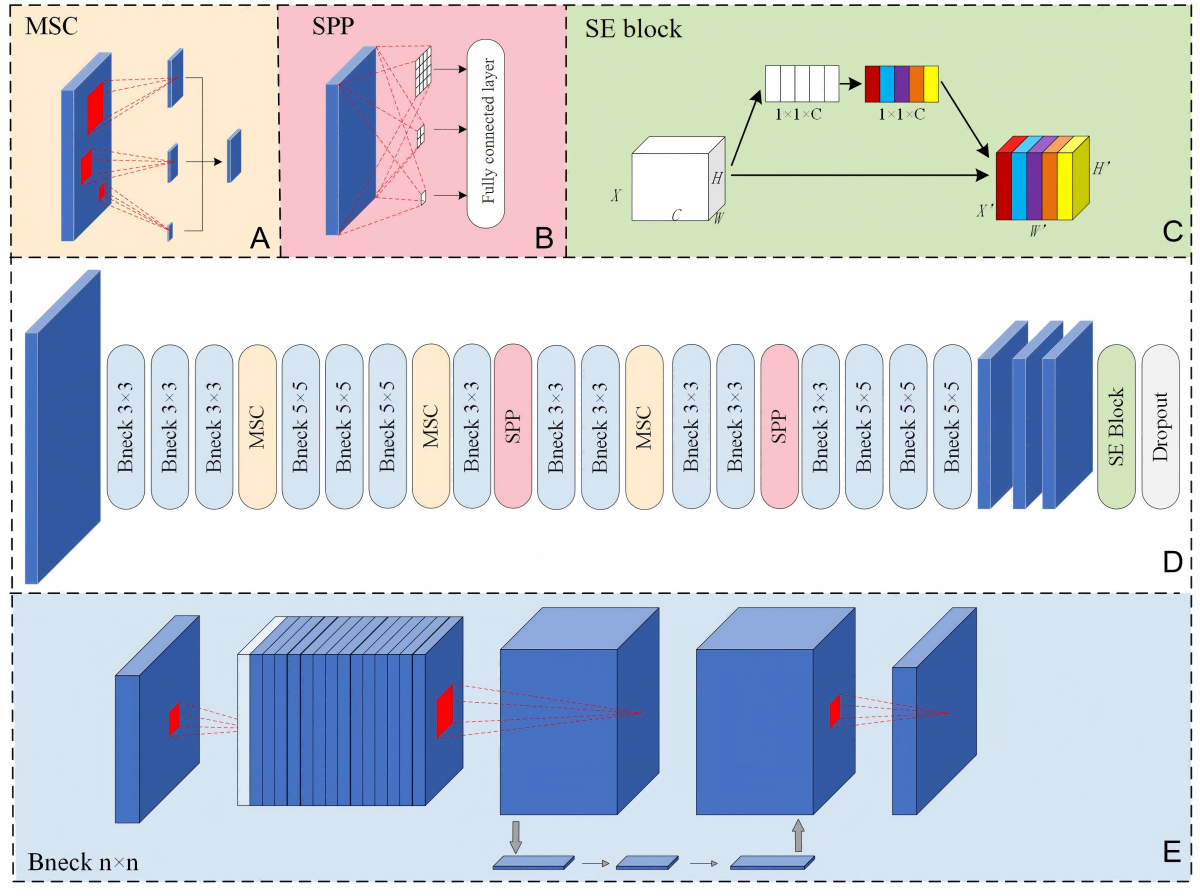
The overall architecture of the proposed model: (A) details of the multiscale convolution (MSC) module; (B) details of the Spatial Pyramid Pooling (SPP) module; (C) structure of the Squeeze-and-Excitation (SE) block module; (D) workflow of the model that included detailed layers of the network; and (E) structure of the inverted residuals module.

We proposed a variant of the MobileNetV3 architecture for classifying TZ, a lightweight convolutional neural network (CNN) specifically designed for efficient operation on portable devices, which was particularly suitable for deployment in resource-limited settings. The overall model structure is presented in Figure 3. Our model retained the depthwise separable convolutions and the HardSwish (H-swish) activation function of MobileNetV3, which reduced computational demand and parameters without compromising accuracy. Multiscale convolution modules have been incorporated into the model to effectively extract features at multiple focal points (Figure 3A), from subtle acetowhite changes in the columnar epithelium to varying TZ-type scopes in the cervix. In addition, we introduced a spatial pyramid pooling module to address features at multiple scales while preserving spatial information in the input images to ensure the richness of feature representation (Figure 3B). The Squeeze-and-Excitation module is a lightweight attention mechanism designed to automatically prioritize the most diagnostically relevant visual patterns within the MobileNetV3 architecture (Figure 3C). In our model, the Squeeze-and-Excitation module was embedded to adjust global feature weights (Figure 3D), enhancing the overall effectiveness of feature extraction. It increased the sensitivity to important features, thereby improving classification performance [18].

### Development of the TZ Segmentation Model

**Figure 4:**
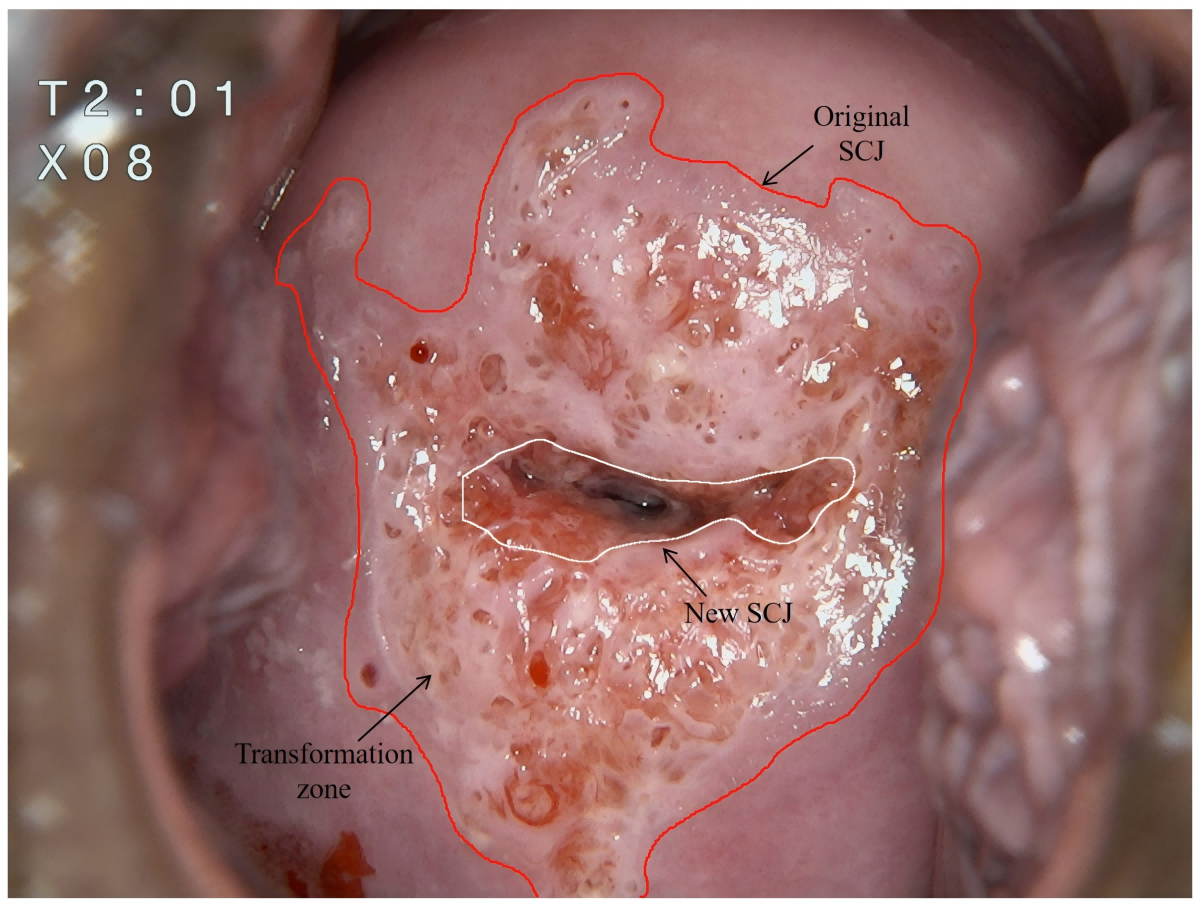
Illustration of the squamocolumnar junction (SCJ) and the transformation zone.

The FastSAM model was used to segment the TZ from the entire cervix in colposcopy images (Figure 2B). FastSAM was fine-tuned for the specific dataset using a YOLO-based framework [19]. Colposcopic images were resized to 640×640 pixels, and the new SCJ was annotated using LabelMe (MIT CSAIL) [20]. By selectively freezing some of the backbone layers of the model, the deeper layers were fine-tuned to improve adaptation to the specific task, without compromising the extraction of general features. The model’s performance was further enhanced by using coarse masks as spatial guides, which allowed the model to focus its attention on the most relevant image regions. The model was trained to delineate the new SCJ, thereby identifying possible TZ areas by this critical boundary. TZ is a dynamic region defined by the area between the original SCJ and the new SCJ. Typically, the original SCJ is considered a virtual line, and identifying the new SCJ is crucial for determining the location of the TZ (Figure 4).

### Software and Hardware

The study was performed on a Windows 11 operating system with an NVIDIA GeForce RTX 3080 8GB graphics card. The models were implemented using Python (version 3.6.13; Python Software Foundation) and the PyTorch (Meta AI) Library (version 1.7.1).

### Evaluation Metrics

Experiment performance of the cervical TZ classification model was evaluated using accuracy, precision, recall, *F*_1_-score, and average precision, as defined in Equations 1-6.

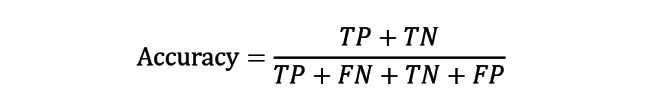

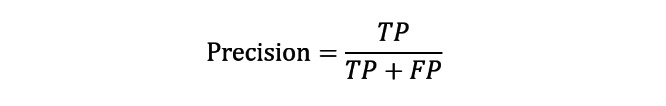

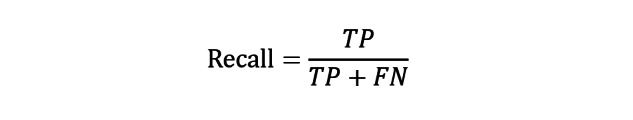

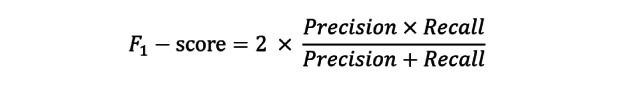

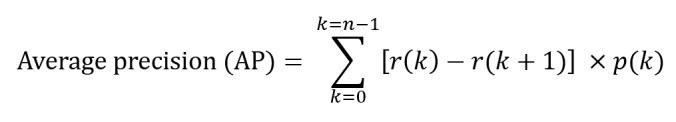

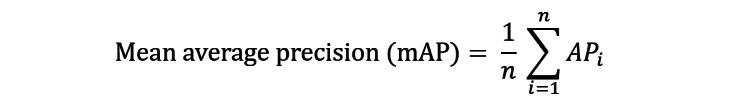













Where TP, FP, and FN denote the true positive, false positive, and false negative predictions, respectively. *r*(*k*) denotes the recall at threshold *k*. *p*(*k*) denotes the precision at threshold *k*, and *n* denotes the number of thresholds. *AP_i_* is the average precision for class *i*, and mAP denotes the mean average precision, where *n* is the number of classes.

### Statistical Analysis

The performance of the classification model was evaluated by comparing it with selected state-of-the-art (SOTA) deep-learning models using metrics such as accuracy, precision, recall, and the area under the precision recall curve, represented by average precision. To evaluate the classification of TZ types, sensitivity, specificity, positive predictive value, and negative predictive value (NPV) were calculated, along with their corresponding 95% CIs. The evaluation metric was defined as the agreement with the expert-provided TZ classification. The demographic characteristics of the study participants were summarized using means and SDs for continuous variables and percentages for categorical variables. A *P* value of less than .05 (two-sided) was considered statistically significant. Statistical analyses were performed using SPSS (version 27.0; IBM Corp) and Python (version 3.7).

### Ethical Considerations

This study was approved by the Institutional Review Board of the Chinese Academy of Medical Sciences and Peking Union Medical College (CAMS and PUMC-IEC-2022-022). Informed consent was not required due to the retrospective nature of the dataset, and all personal information and images were completely anonymized. We commit that all research data will be used for academic research purposes.

## Results

### General Information of the Study Dataset

**Figure 5:**
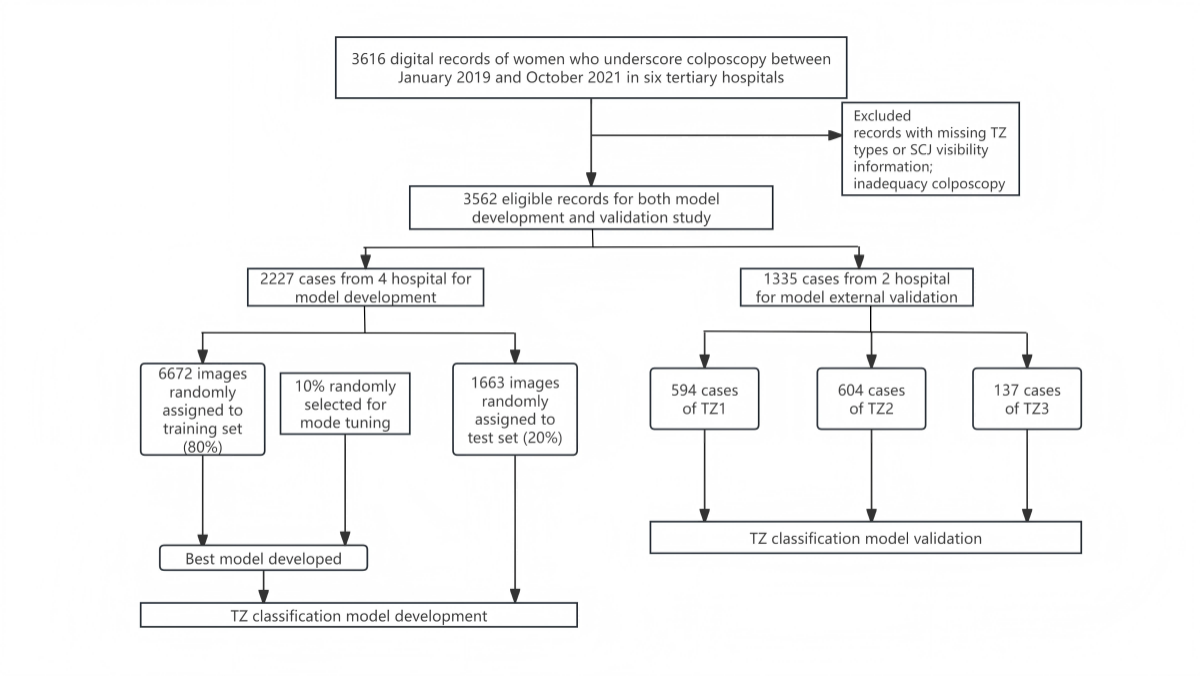
Flowchart of the study case collection. SCJ: squamocolumnar junction; TZ: transformation zone.

For the classification modeling, 8335 colposcopy images from 4 hospitals were selected for training and evaluation. These images included consisting of 2788 images of TZ1, 2663 images of TZ2, and 2884 images of TZ3. In the external validation study, 1335 cases were selected from 2 hospitals for model inference to predict the TZ types (Figure 5). The demographic characteristics of each participant, including age, colposcopic findings, and the distribution of each TZ type, are provided in Table 1. TZ types were found to be significantly associated with the participants’ age group distribution (*P*<.001). The mean age of all participants at the time of colposcopy was 37.87 (SD 9.99) years, with the mean age of the TZ3 group being 45.04 (SD 11.69) years.

**Table 1:** Demographic characteristics and distribution of cervical TZ^a^ types. Differences were analyzed using the chi-square test.

Characteristic					TZ1 (n=594)	TZ2 (n=604)	TZ3 (n=137)	*P* value	
**Age group (year), mean (SD)**					34.44 (8.45)	39.62 (9.64)	45.04 (11.69)	<.001	
	24-29				140 (23.6)	62 (10.3)	10 (7.3)		
	30-49				386 (65.0)	432 (71.5)	79 (57.7)		
	50-65				68 (11.4)	110 (18.2)	48 (35.0)		
**Parity, n (%)**									<.001
		0			159 (26.8)	95 (15.7)	18 (13.1)		
		1-3			428 (72.1)	488 (80.8)	116 (84.7)		
		>3			7 (1.2)	21 (3.5)	3 (2.2)		
**Menstrual status, n (%)**									<.001
			Menopause		25 (4.2)	71 (11.8)	43 (31.4)		
			No menopause		569 (95.7)	533 (88.2)	94 (68.6)		
**SCJ^b^ visibility, n (%)**									<.001
			Completely visible		594 (100.0)	73 (12.1)	0 (0)		
			Partially visible		0 (0)	531 (87.9)	28 (20.4)		
			Invisible		0 (0)	0 (0)	109 (79.6)		
**Pathologic diagnosis, n (%)**									<.001
				Normal or benign	220 (37)	188 (31.1)	26 (19)		
				CIN^c^1	220 (37)	213 (35.3)	51 (37.2)		
				CIN2	124 (20.8)	131 (21.7)	23 (16.8)		
				CIN3	24 (4)	63 (10.4)	13 (9.5)		
				Cancer	6 (1)	9 (1.5)	24 (17.5)		

^a^TZ: transformation zone.

^b^SCJ: squamocolumnar junction.

^c^CIN: cervical intraepithelial neoplasia.

### TZ Classification Results

Cervix detection with a bounding box was used for feature engineering. Proper cervix extraction improved classification accuracy. A total of 8335 cervix images were investigated and resized to 224×224 pixels before being input into the classification model. Out of these, 1663 images were used to evaluate the model’s performance.

**Figure 6:**
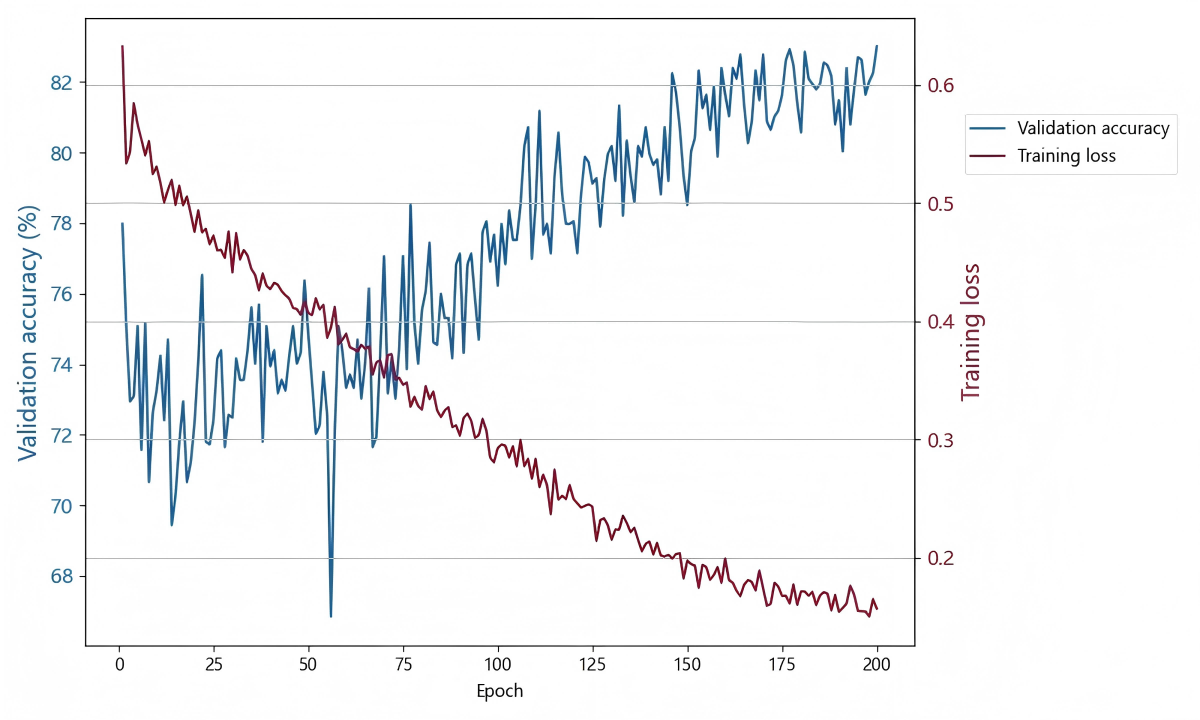
Validation accuracy and training loss curve for the proposed classification model.

Around 80% of the images were randomly selected as the training set, with the optimal weight parameter model selected during training being used to classify the images in the test set. The validation accuracy of the model gradually improved as the number of epochs increased, as shown in the validation plot (Figure 6). After approximately 100 epochs, both validation accuracy and training loss stabilized, reaching a peak validation accuracy of 83% at the 200th epoch. The validation accuracy fluctuated significantly during the first 25 epochs, it showed a general upward trend, while training loss rapidly decreased, indicating active learning and adjustment of the model. The model reached an optimal balance point and performed well after the 75th epoch when validation accuracy stabilized and training loss continued to decrease but with minimal changes. Overall, the model demonstrated rapid convergence in the early stages of training, with steady performance improvements in the later stages.

Our proposed model accurately classified cervical TZ types, as shown in Table 2. The highest classification accuracy of 83.97% and precision of 83.93% were achieved on the test set. For the 3 TZ types, the sensitivity was 84.74% for TZ1, 78.95% for TZ2, and 87.87% for TZ3, while the specificity was 89.99% for TZ1, 91.98% for TZ2, and 94.03% for TZ3. The detailed values for sensitivity, specificity, positive predictive value, and NPV are presented in Table 3. According to the classification performance of TZ types, the sensitivity and NPV for TZ2 were significantly lower.

**Table 2:** Comparative performance results of the proposed classification model with other state-of-the-art models.

Model	Accuracy (%)	Precision (%)	Recall (%)	*F*_1_-score (%)	mAP^a^ (%）
ResNet50	59.22	57.84	56.89	54.66	63.13
VGG16	70.33	69.76	69.21	69.19	74.64
ViT	59.46	57.64	57.39	56.49	63.76
EfficientNet	62.06	60.97	61.18	60.66	63.63
ShuffleNet	74.47	73.9	73.67	73.66	80.98
MobileNetV3	75.06	74.49	74.64	74.55	82.72
Proposed model	83.97	83.93	83.85	83.86	92.17

^a^mAP: mean average precision.

**Table 3:** Classification performance of the proposed classification model on the test set.

TZ^a^ types	Sensitivity (%)	Specificity (%)	PPV^b^ (%)	NPV^c^, (%)	Average precision (%)
TZ1	84.74	89.99	80.96	92.15	91.84
TZ2	78.95	91.98	82.19	90.30	89.06
TZ3	87.87	94.03	88.64	93.60	95.62

^a^TZ: transformation zone.

^b^PPV: positive predictive value.

^c^NPV: negative predictive value.

The SOTA deep-learning networks for interpreting colposcopy images were selected to train on our dataset for comparison with our model. Table 2 presents the experimental results of the test set based on various evaluation metrics. The traditional ResNet50 and Vision Transformer models performed poorly in terms of accuracy and precision, achieving only 59.22% and 57.84%, and 59.46% and 57.64%, respectively. VGG16, with its relatively low-density network structure, performed better than ResNet50 in feature extraction. In addition, we selected 2 lightweight networks for comparison, EfficientNet and ShuffleNet, with higher accuracy of 62.06% and 74.47%. The proposed model achieved the highest accuracy of 83.97% among SOTA models.

### TZ Segmentation Result

**Figure 7:**
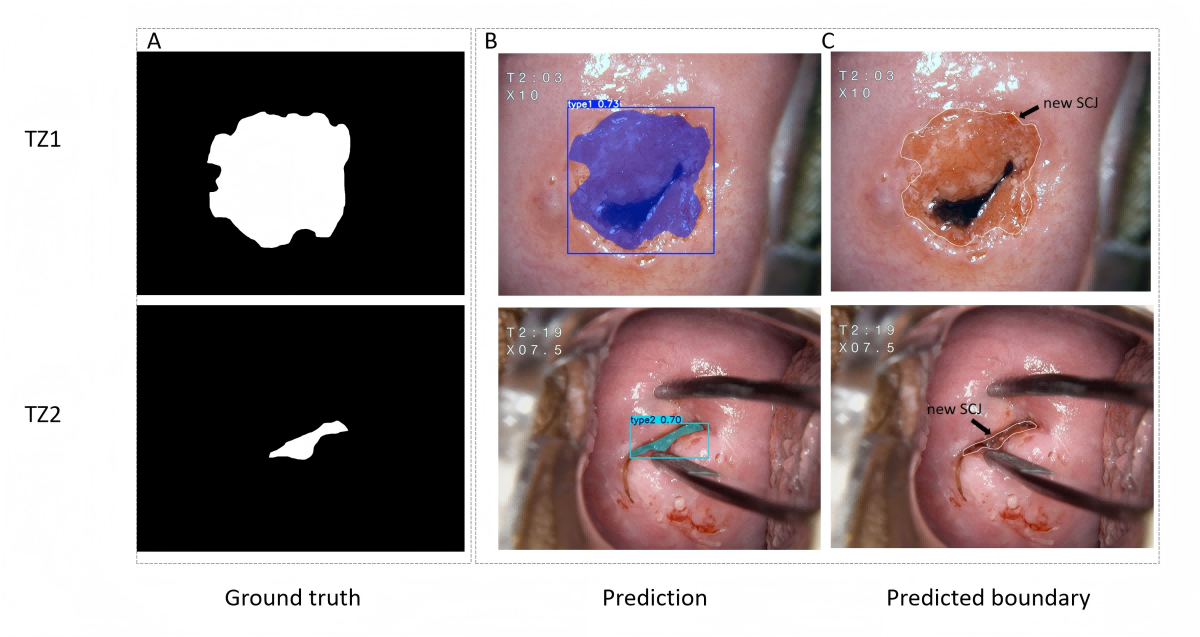
The segmentation model inference process. (A) Ground truth of the polygonal new SCJ outline. (B) Predictive segmentation of the out-of-TZ area. (C) Representative prediction of the new SCJ guidance line. SCJ: squamocolumnar junction; TZ: transformation zone.

To enhance AI’s clinical guidance, we used a pretrained FastSAM model to segment the TZ region by visualizing the new SCJ. A portion of the training data was used to annotate the new SCJ (Figure 7A), excluding TZ3, as the new SCJ is located in the endocervix. The segmentation model predicted the negative foreground region indicating the area outside the target TZ region (Figure 7B). The region boundary of the mask prediction was the predicted new SCJ (Figure 7C). In the test set, the overall recall and mAP50 (mean average precision at 50% Intersection over Union) of predicted mask were 0.78 and 0.75, respectively.

### Validation Results

**Figure 8:**
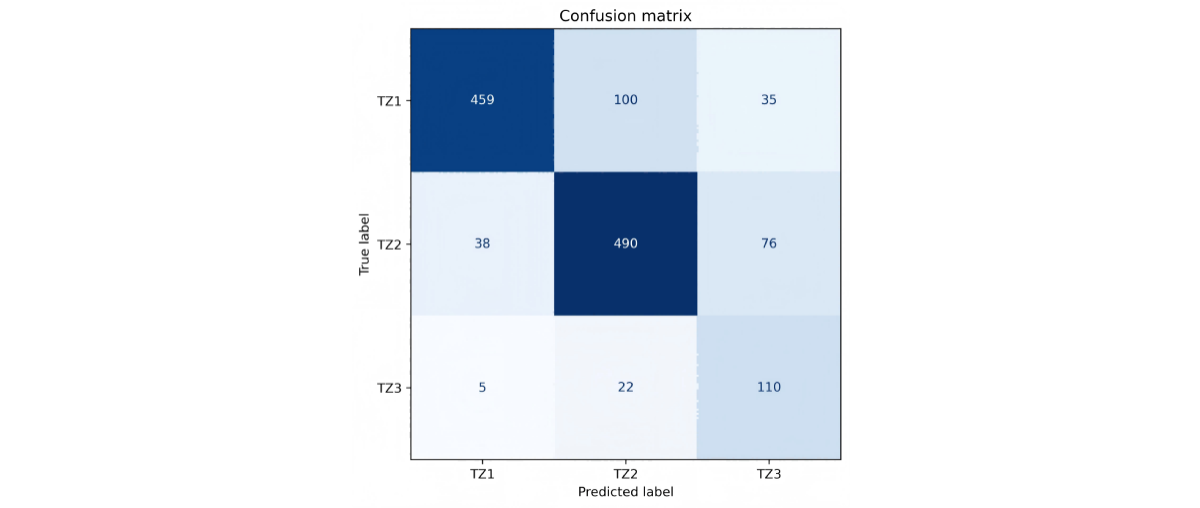
Confusion matrix of the proposed classification model in validation. TZ: transformation zone.

An independent dataset of 1335 cases was used to evaluate the generalization of the TZ classification model, distributed as 594 cases of TZ1, 604 cases of TZ2, and 137 cases of TZ3. The overall classification accuracy was 79.33%. The model’s predicted sensitivity for TZ1, TZ2, and TZ3 was 77.3% (95% CI 73.9%-80.6%), 81.1 (95% CI 78.0%-82.3%), and 80.3% (95% CI 73.7%-86.9%), respectively, while the specificity was 94.2% (95% CI 92.4%-95.8%), 83.3% (95% CI 80.6%-85.9%), and 90.7% (95% CI 89.1%-92.3%). The classification performance is presented in Figure 8 and Table 4.

**Table 4:** Classification performance on the validation dataset.

TZ^a^ types	Sensitivity (%; 95% CI)	Specificity (%; 95% CI)	PPV^b^ (%; 95% CI)	NPV^c^ (%; 95% CI)	*F*_1_-score (%)
TZ1 (n=594)	77.3 (73.9-80.6)	94.2 (92.4-95.8)	91.4 (88.9-93.8)	83.8 (81.3-86.3)	83.76
TZ2 (n=604)	81.1 (78.0-82.3)	83.3 (80.6-85.9)	80.1 (76.8-83.2)	84.2 (81.5-86.9)	80.59
TZ3 (n=137)	80.3 (73.7-86.9)	90.7 (89.1-92.3)	49.8 (43.0-56.7)	97.6 (96.7-98.5)	61.45

^a^TZ: transformation zone.

^b^PPV: positive predictive value.

^c^NPV: negative predictive value.

## Discussion

### Principal Findings

In this study, we proposed a classification and identification model that facilitates clinical colposcopy examinations of cervical TZ. Our AI model achieved an accuracy of 79.33% in the task of classifying the three types of TZ in the external dataset. The sensitivity of TZ3 was 80.3%, and the specificity was 90.7%, which were satisfactory. TZ3 could be accurately differentiated, which further decreased the chance of missed diagnoses of high-grade lesions [21], and it was valuable for guiding ECC and recommending appropriate treatments. The AI model had increased sensitivity on TZ2 and lower sensitivity on TZ1 compared to the performance on the test set, which might be attributed to the similarity between TZ1 and TZ2. According to the standard terminology of the IFCPC, TZ1 and TZ2 were both visible either with or without the assistance of an endocervical speculum [22]. TZ2 was nearly entirely visible in some cases, while the SCJ was only visible at the endocervical canal’s border. Therefore, it was difficult to differentiate it from TZ1, which frequently required expert evaluation in strict accordance with established guidelines. In clinical practice, the treatment management for TZ1 and TZ2 is generally similar [6]. Our AI model demonstrated noninferior performance in classifying them and could be used to assist less experienced or inexperienced colposcopists in the colposcopic examination process. The overall model accuracy from the validation study was similar to the performance during model training, indicating that it is capable of generalizing fairly well.

### Comparison With Other Studies

In terms of AI techniques, we proposed a method based on the variant MobileNetV3 architecture for cervical TZ classification and FastSAM for segmenting TZ in colposcopy. Currently, only a few deep learning–based models have been developed to classify cervical TZ from colposcopy images. Dash et al [23] conducted a TZ segmentation and classification model based on colposcopy images from the IARC image bank. Similarly, Cao et al [24] developed a high-performance, deep learning model based on retrospective image data collected from one hospital [24]. Comparatively, our method was based on a multicenter study with a more diverse dataset of colposcopy images. While this diversity added to the challenge of feature extraction, the classification model showed better accuracy. Second, the proposed segmentation model could precisely annotate the new SCJ and indicate the approximate location of TZ, which could greatly assist junior colposcopists in selecting biopsy sites. Effective biopsy prioritization focused on the most severe lesions, particularly those within the TZ. Compared with the previous study [23], our method delineated a new SCJ rather than the original SCJ, which can provide more effective assistance and insights for colposcopic clinical examination and treatment. Furthermore, conducted external validation for the TZ classification model for the first time, and our model demonstrated strong stability and generalization performance.

### Clinical Implications

In resource-limited settings, colposcopists demonstrated significantly lower clinical skills and performance than those observed in our study. The results of a study evaluating the colposcopic abilities of colposcopists in underserved Chinese communities indicated that colposcopists had a mere 22% accuracy specifically for TZ3 [8]. Similarly, in another study, colposcopists’ clinical diagnostic abilities were assessed before and after intensive training [9]. It was found that junior colposcopists had only 49.1% accuracy in classifying TZs. Despite the notable improvement, their accuracy remained below optimal levels at 68.6% following training. In addition, a comparable study conducted in Europe demonstrated that junior colposcopists were only able to detect TZs with a 55% accuracy rate [25]. According to these findings, colposcopists at less-experienced levels generally exhibited inadequate colposcopic performance. However, AI might offer a promising solution to enhance colposcopic capabilities and clinical decision-making confidence. Compared to these studies, our TZ classification model demonstrated strong performance with a high accuracy of 83.97% in the test set and achieved much higher sensitivity in predicting three TZ types in the validation study. Based on the results of previous studies, our model was more effective at stand-alone classification than that of junior colposcopists. Therefore, the method presented in this study accurately identifies TZ in colposcopy images, providing a valuable reference for colposcopists when making clinical decisions. The findings from this study supported the potential of the proposed AI-based TZ identification method as a promising adjunct tool for colposcopic examinations, particularly when integrated with AI colposcopic diagnostic systems and digital colposcopes. Dynamic digital imaging with AI assistance enhances the objectivity of colposcopic examinations and might address the diagnostic subjectivity of less experienced colposcopists. When AI-guided digital colposcopists are deployed in resource-limited health care settings, colposcopists will be able to receive intuitive and accessible guidance on clinical features from AI during the colposcopic examination in real time, supporting less-experienced colposcopists in improving their overall colposcopy skills. Combining AI results with colposcopist assessments helps reduce diagnostic bias, improve colposcopic examination capability, and narrow the gap with resource-rich areas. It addresses the minimization of missed diagnoses, the facilitation of early detection of lesions, the reduction of the risk of CIN progression, and the reduction of the burden of cervical cancer [26]. The application of AI will be integral to improving the quality of colposcopy services in low-resource settings.

The development and application research of AI-guided colposcopy models has emphasized their auxiliary value in clinical practice [14,27]. With the advent of innovations in AI algorithms, these models rely predominantly on mainstream CNNs coupled with transfer learning, as a method of unsupervised learning for image feature learning [28,29]. In some AI models, object detection is used as a metric for identifying lesion areas; however, it remains limited to binary classification tasks. Nevertheless, the multiclassification of normal cervix, low-grade squamous intraepithelial lesion, and high-grade squamous intraepithelial lesion plus poses significant challenges to these models. A clinical perspective suggested that acetowhite changes within TZ have a higher likelihood of association with CIN lesions than those outside of TZ. While the lesion extends across the new SCJ, a biopsy should be taken within the TZ or at the new SCJ. Therefore, by incorporating the TZ-type information as a weight in the model, the AI could be able to perform a more effective feature engineering process within the TZ. It is anticipated that the AI model will markedly enhance the capability of lesion detection, thus eliminating the current limitation in which precise identification is confined to CIN2+ lesions. Our proposed AI system delineated the new SCJ on high-resolution colposcopy images, and it enabled the approximate location of the TZ to be visualized more intuitively, thereby guiding more effective colposcopy examination and biopsy procedures.

For applying AI techniques in limited-resource settings, we proposed a model based on the MobileNetV3 architecture for cervical TZ classification in colposcopy images. Since Google introduced MobileNet in 2017 [30] as a lightweight CNN architecture, it has gained significant attention for its efficiency and accuracy. A lightweight AI model is more energy efficient and requires significantly fewer computational resources than a large-scale AI model, aligning with sustainable AI practices. It has been designed to operate efficiently on battery-operated devices, making it especially suitable for deployment in remote regions with limited power availability and internet access. By minimizing the reliance on high-cost hardware and extensive cloud infrastructure, lightweight AI models enable resource optimization in low-resource settings and facilitate equitable access to AI. Our AI model achieved efficient computation and robust classification performance on portable devices and can be applied actively in a variety of clinical settings to validate its generalization ability. A further economic evaluation is required to support the decision to adopt novel technologies in screening strategies.

### Limitations and Future Directions

There are several limitations to this study. First, the scale of the dataset we included was limited, which restricted the ability to support training deeper or more complex deep neural networks. However, the quality and standardization of image acquisition were assured, and the appropriate network depth was chosen to maximize feature extraction. It is necessary to obtain more high-quality colposcopy images with endocervix expansion by auxiliary instruments per case to train more complex networks that can extract additional features and potentially improve classification accuracy in TZ2 and TZ3. To address the suboptimal performance in TZ2 classification, expanding the dataset for TZ2 in future studies could enable the model to capture more distinct texture features that differentiate it from TZ1 and TZ3. The model could be further enhanced by applying edge detection techniques in regions where it is difficult to distinguish TZ2 from TZ3 and to highlight subtle morphological changes at the new SCJ. Second, the external validation was limited to evaluating the AI results alone. Further research is needed to assess the impact of AI-aided colposcopists in the classification of TZ. A prospective clinical trial is further needed to investigate whether the method can be applied to real-world colposcopy clinics. In addition, although we collected the validation datasets from two different hospitals, the scale of the external validation dataset is relatively limited, particularly when it comes to representing clinical scenarios that involve different colposcope vendors and lighting conditions. Future studies will focus on expanding both the size and scope of the dataset, incorporating data from more regions for more comprehensive validation. Furthermore, transfer learning or domain adaptation techniques may be used to improve the model’s robustness and adaptability to various imaging conditions. Finally, the TZ classification lacks an absolute subjective gold standard but is guided by colposcopic examination criteria and expert consensus. However, the colposcopy expert panel from tertiary hospitals conducted the “ground truth” categories for this study in accordance with established guidelines from IFCPC. In light of the subjectivity inherent in TZ classification, using only single-image modality data for model development is limited. Model optimization in the future may implement multimodal learning, which incorporates image data with associated clinical factors (eg, age, menstrual status, gravidity, and parity) to reduce the subjectivity of the model and enhance TZ feature discrimination. Furthermore, the construction of a knowledge graph based on existing consensus and colposcopic examination guidelines will help the model adhere to various rules or logic during the learning process.

### Conclusion

In this study, an accurate AI-based method was developed and evaluated for automatically identifying cervical TZ using colposcopy images. The proposed method was the first application of a lightweight CNN for cervical feature extraction and applied a general segmentation model for TZ delineation among multicenter images, achieving commendable classification accuracy on TZ. The proposed approach has the potential to adapt to various colposcopy clinical environments and improve AI-guided colposcopy practice.

## Abbreviations

AI: artificial intelligence
CIN: cervical intraepithelial neoplasia
CNN: convolutional neural network
ECC: endocervical curettage
IFCPC: International Federation for Cervical Pathology and Colposcopy
mAP50: mean average precision at 50% Intersection over Union
NPV: negative predictive value
SCJ: squamocolumnar junction
SOTA: state-of-the-art
TZ: transformation zone

## Data Availability

The datasets generated or analyzed during this study are not publicly available due to data-sharing policies set by the funding institution but are available from the corresponding author on reasonable request.
